# Micro-Structure Modelling and Electrical Properties Analysis of PZT Matrix Ferroelectric Composites

**DOI:** 10.3390/ma13020448

**Published:** 2020-01-17

**Authors:** Weibin Zhou, Jinbo Fan, Zhenchao Xin, Guodong You

**Affiliations:** School of Electronic Information and Automation, Tianjin University of Science and Technology, Tianjin 300384, China; fanjinbo94@163.com (J.F.); xinzhenchao2014@163.com (Z.X.); yougdong@tust.edu.cn (G.Y.)

**Keywords:** 3D-model, composite, dielectric constant, random close packing of spherical, finite element method

## Abstract

PZT matrix ferroelectric composite is an important research topic in material science because of its many practical, industrial, and scientific applications. Materials with high dielectric permittivity are used to manufacture electronic devices, particularly capacitors and dynamic random access memory (DRAM). Therefore, the development of reliable and efficient micro models to be utilized in analyzing electrical properties can be of great value in accelerating research in this field. In this paper, a 3D microstructure model for PZT matrix ferroelectric composites has been developed and adopted the finite element method (FEM) to calculate the dielectric constant. The microscopy parameters of developed microstructure model are acquired based on the real composites from X-ray (micro-) diffraction and stereological method. The dielectric constant of different volume ratios of PZT matrix ferroelectric composites can be calculated by accurately controlling the volume of Ferrite particles. At the point of validation, the proposed approach makes visual and numeric comparisons between the morphology of the real microstructure and the model generated by the proposed technique. The simulation results by our method was essentially in agreement with experimental results in other literature. Simulation Experimental results also demonstrate that the dielectric constant of PZT matrix ferroelectric composites is significantly changed while the volume ratio of high dielectric phase particles was below 20%. PZT matrix ferroelectric composites Consequently, this method can be easily extended to composites preparation.

## 1. Introduction

A strong dependence between macroscopic properties and micro-structures occurring in polycrystalline materials means that these materials are intensely investigated in various scientific domains [1,2,3]. Piezoelectric ceramic (PZT) has the advantage of promising and robust ferroelectric and piezoelectric properties and it has become extensively applied in electronic industries, such as switching devices, super-capacitors with high energy density characteristics, pyroelectric elements and sensors, micro-electromechanical transducers, piezoelectric sensors and actuators. There are some reports for improving the dielectric properties of the PZT by doping or making composites. An improvement in the properties of PZT has been reported by doping the series of materials such as Nb, Fe, and Mn [4,5]. If the two materials can be combined into dual-phase composites that have the advantages of two materials, the composites have great importance in the potential engineering application point of view where they are used in many electrical devices [6,7,8,9].

However, the main disadvantage of the techniques based on sample preparation and detection results is the complex and time-consuming post-processing required to analyze the amount of data produced. Relying upon the destructive data acquisition methods cannot furbish simulations of the physical response that can be compared against experiments since the specific sample was destroyed during data acquisition on the microstructure itself. In recent years, the introduction of computational simulation techniques has reduced the computational cost and time of the procedure [10,11,12,13,14]. Differing from a physical experiment, the method provides simulation using random numbers to conduct many virtual experiments on a computer and then analyzes the results of these experiments on a statistical basis to draw conclusions. Random close packing of spherical (RCPS), which is confirmed to be an effective technique, has been proven to be widely used in computer simulation [15,16]. Therefore, the model of PZT matrix ferroelectric composites is built by RCPS.

It is well-known that numerical simulations based on the finite element method (FEM) are effective techniques for performing the analysis of properties for composites [17,18,19,20,21], such as Guo, XG, et al., who established a molecular dynamics (MD) simulation model of high chromium alloy based on alloy structure by using atomic random replacement and two bubble algorithms. Thus, the properties of the high chromium alloy model are improved, and it becomes more suitable for precision machining [22]. Sheikholeslami, M uses the finite element method to simulate the unstable process of a complex shape energy storage box. It is concluded that a higher heat transfer rate can be obtained by using nano-reinforced phase change material (NEPCM) instead of pure phase change material (PCM) [23]. The hereby work is focused on polycrystals particles of PZT matrix ferroelectric composites. The numerical method, allowing to calculating dielectric constant on the basis of simulations performed for synthetic microstructures, is proposed for these materials.

In particular, the ability to accurately reproduce microstructural features is fundamental for the valid evaluation of the electrical properties’ finite element method of PZT matrix ferroelectric composites. To illustrate the effectiveness of the proposed method, the approach presented in this paper relies solely upon the data collected from the 2D scanning electron microscope (SEM) images of polycrystalline microstructures. The effects of the volume ratio of high dielectric phase particles on the electrical properties of dual-phase composites are investigated. The capability of the simulation results is verified with the results of the experiment available in other literature.

## 2. Simulation Process

### 2.1. The Overall Approach

All the programs in this paper are implemented through MATLAB and TetGen. To evaluate PZT matrix ferroelectric composites’ dielectric constant, the work is divided into four parts, including generating 3D random close packing of spheres, generating grain structure, automatic mesh subdivision and finite unit calculation. The macroscopic dielectric constant of PZT matrix ferroelectric composites is mainly determined by their microstructure and the volume ratios of dual-phase materials. Therefore, the first step is setting the inputs, according to X-ray (micro-) diffraction microscopy provides 3D microstructural phase representations, which contain the number of phases. In addition, the grains of polycrystalline composites have been shown to obey lognormal distribution [24]. In previous work, an effective computer numerical method was established to determine the continuous numerical empirical probability function of the 3D distribution of spherical two-phase particles [25]. Sequentially, the grain model after edge cutting is obtained by counting the high-low dielectric phase grain element distribution of the RCPS model and adjusting boundary conditions.

After that, the grain model is an adaptive subdivision by TetGen software. The data is loaded to MATLAB to process. The characteristics of finite element units are determined by the processed data.

Then, the PZT matrix ferroelectric composites’ dielectric constant is carried out to evaluate adopted the finite element method. According to the minimum energy rule, the macroscopic dielectric constant is obtained by reassembling the finite element units and calculating. Finally, the macroscopic dielectric constant under different ratios of dielectric constant can be obtained.

By changing the ratios of the second phase particles in ferrite/PZT dual-phase composite, its electrical properties can be controlled which shows satisfactory behavior on different applications.

### 2.2. Generating 3D Random Close Packing of Spheres

In order to establish a three-dimensional microscopic model and ensure the agreement between the model and the PZT matrix ferroelectric composites, the random close packing of spheres (RCPS) is chosen to use to form the microscopic basal model. The RCPS model, a basic geometric model, has provided great help in many physical and engineering problems and played a guiding role in the field of materials [26,27,28]. The RCPS method is a modified rearrangement algorithm, and there is another method called the sequential generation method. The electrical properties of the simulation model vary with the method of the duty ratio per unit volume, the radius of the generated sphere and distribution pattern [29,30].

**Figure 1:**
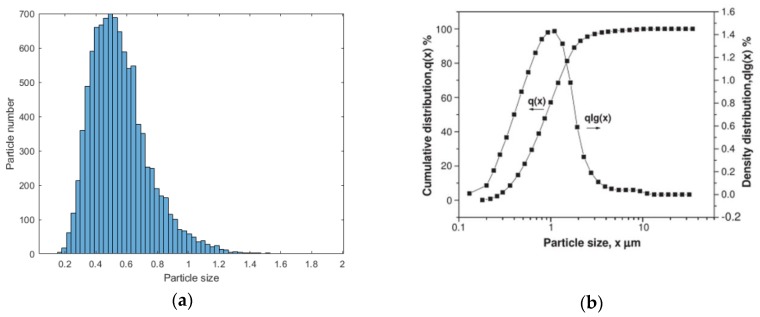
(**a**) The particle size distribution of model for PZT matrix ferroelectric composites. (**b**) The particle size distribution of the PZT (piezoelectric ceramic) powder [31].

Thus, according to X-ray (micro-) diffraction, microscopy provides 3D microstructural phase representations and there is no intermediate or interfacial phase in PZT matrix ferroelectric composites [3,28,31]. Furthermore, by controlling the above-mentioned conditions, the distribution of our simulation model is in agreement with real PZT matrix ferroelectric composites. The particle size distribution generated is shown in Figure 1.

**Figure 2:**
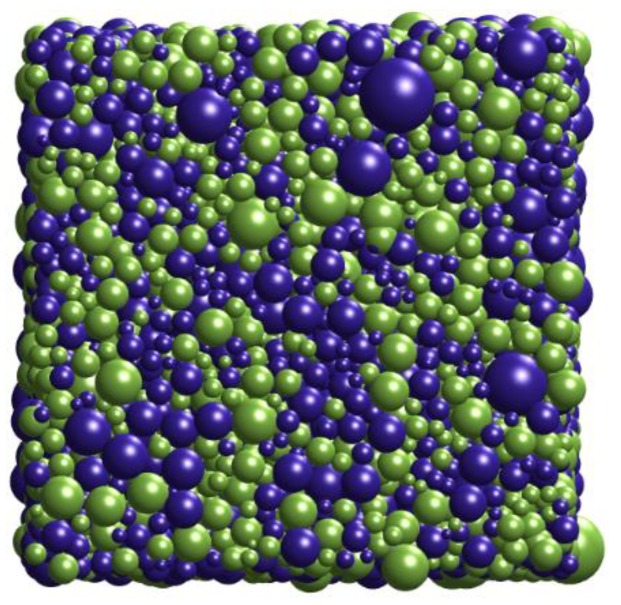
The RCPS (random close packing of spheres) model of PZT matrix ferroelectric composites (blue particles are PZT, and green particles are ferrite).

The generated RCPS model is shown in Figure 2.

### 2.3. Generating Grain Structure 

After getting the appropriate RCPS model, the Laguerre Voronoi method is used to generate a grain model whose volume fraction could be controlled accurately. To form a grain model without voids, the Laguerre Voronoi method is adopted to distribute the voids of the RCPS model to each grain. The grain model completely inherits the phase volume ratios of the RCPS model.

The irregular boundary can affect the adaptive mesh subdivision and change the volume fraction of the second-phase particle in this model. In order to eliminate the boundary influence of the 3D microstructure model, it is necessary to cut the edge of the model for forming the model of ferrite/PZT dual-phase composite after getting the appropriate 3D microstructure model.

The count method is as follows:

First, the grains are divided into two types: Pa phase grain and Pb phase grain. Next, the total volume of two kinds of grains needs to calculate, called Vt. The total volume of Pa phase, called Va. The total volume of Pb phase, called Vb.

If the ideal volume ratio of high to low dielectric phase grains is Vhigh: Vlow, the total volume of Pa phase grains at this time should be:

(1) Va=VtVhigh/(Vhigh+Vlow).

At this point, the total volume of Pb phase particles should be:

(2) Vb=Vt Vlow/(Vhigh+Vlow).

**Figure 3:**
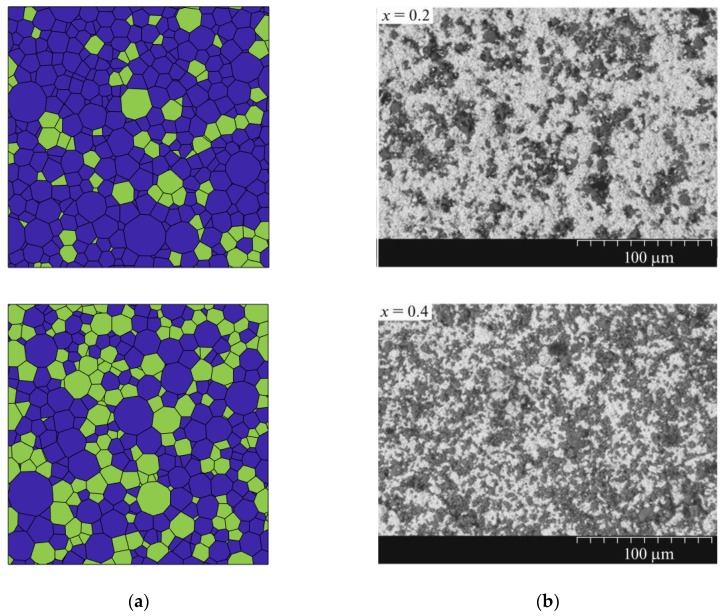
(**a**) The grain model of PZT matrix ferroelectric composites (blue grains are PZT, and green grains are ferrite). (**b**) SEM photos of PZT ceramics and composites of different compositions (light grains are PZT, and dark grains are ferrite—X is the proportion of ferrite volume) [31].

By calculating, it can ensure the grains have the same volume ratio as well as RCPS models. The space gap between spheres in RCPS, which is filled when the model of PZT matrix ferroelectric composites formed. In order to make sure of the validity of this model, it’s necessary to compare real images of SEM with the model in Figure 3. At this time, the model has begun to take shape, and the grain structure of PZT matrix ferroelectric composites can be seen intuitively at different volume ratios.

### 2.4. Automatic Mesh Subdivision

The model established is a three-dimensional model, and the basic unit of three-dimensional division can be divided into three-dimensional tetrahedral units, pentahedral units, hexahedral units, and so on [32,33,34,35]. The grain model established has many irregular polyhedral bodies and many sharp angles before segmentation, so three-dimensional tetrahedral units is chosen to divide them. In order to facilitate subsequent calculation, TetGen software is chosen to achieve the adaptive subdivision of the model.

**Figure 4:**
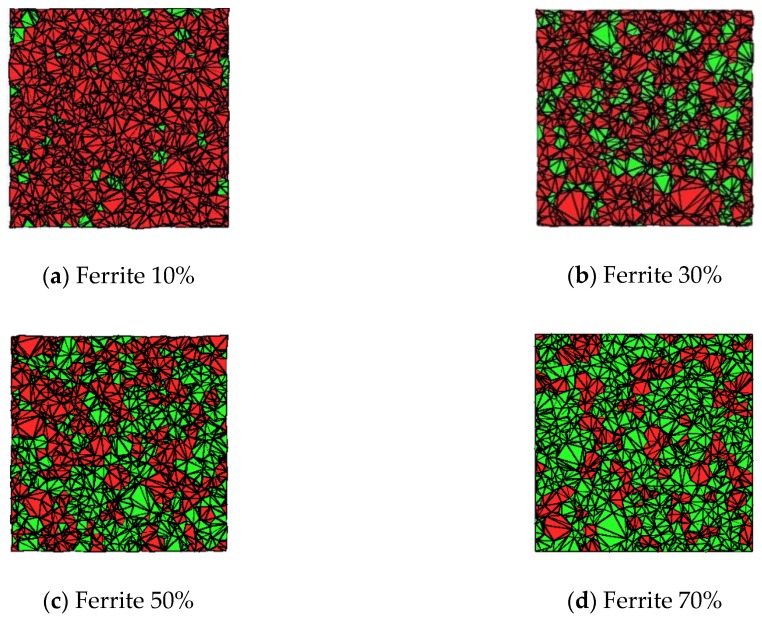
The grain structure of PZT matrix ferroelectric composites with different volume ratios after subdivision. (red grains are PZT, and green grains are ferrite).

Figure 4 shows that after subdivision of different ferrites volume proportion, in which red particles are for ceramic matrix and green particles represent ferrites. It’s clearly found that all basic units are tetrahedron.

### 2.5. Finite Unit Calculation

**Figure 5:**
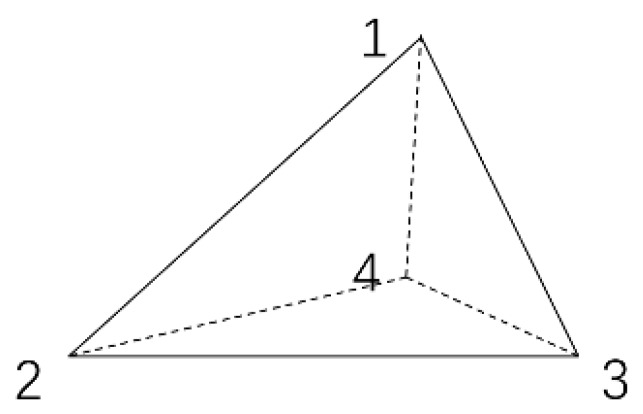
Tetrahedral unit.

The using basic unit is a three-dimensional tetrahedral unit, as Figure 5 shows, so the finite element adopted is also a four-nodes tetrahedral unit. For the convenience of description, it’s better to choose the center of the tetrahedral shape to locate the origin of the local coordinate. The nodes and coordinates of four corners are recorded as Angle1 (x1, y1, z1), Angle2 (x2, y2, z2), Angle3 (x3, y3, z3), Angle4 (x4, y4, z4).

There are two angular nodes on each side. The potential function is linearly distributed on each side, and the tetrahedron of the function also must be linear on each face. In summary, here is the following potential function which is approximated by interpolation functions:

(3) $$U=A_{1}+A_{2}x+A_{3}y+A_{4}z.$$

By transforming it into matrix form and using Cramer’s Rule, $A_{1},\;A_{2},\;A_{3},\;A_{4}$ in the potential function can be obtained.

At this point, the coordinates of each node on the tetrahedral element could be calculated and $A_{1},A_{2},A_{3},\;A_{4}$ can be obtained with the potential function. In other words, all the potentials in a tetrahedral element can be obtained by the potentials of four nodes, so the next step is to obtain the potential values of all unknown nodes.

We need to define the characteristics of the units, this paper uses the energy minimum method, so the first calculation is the energy in each unit.

According to the energy of Poynting theorem, in a three-dimensional static electric field, the energy of the electric field is as follows:

(4) $$W=1/2\;\varepsilon \int \int \int {\left|E\right|}^{2}\;\mathrm{dV},$$

where ε is the dielectric constant of the electric field acting area, E is the electric field strength of the area, and the relation with the electric potential U is as follows:

(5) E=−∇u

(6) $$W^{e}=1/2\cdot U\times S\times U^{T}$$

where S is a differential matrix for the vertices of a tetrahedron.

After obtaining the energy of each tetrahedral unit and assembling all the units together, the total energy of the established model can be obtained, denoted as W. In this paper, the description of the total energy W of the model is the total energy of solving the regional electric field, which can be regarded as the sum matrix of each unit matrix—that is to say, the U matrix and S matrix described in Equation (8). U matrix is the potential matrix, which is composed of the node potential of all tetrahedral units, and the S matrix is composed of the $S^{e}$ matrix of each unit in the model.

**Figure 6:**
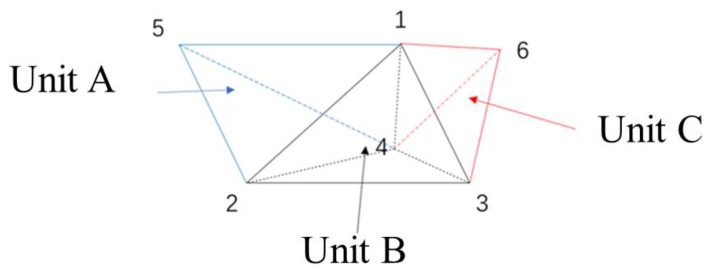
A model consisting of three tetrahedral units.

As shown in Figure 6, the small model consists of three tetrahedral units. For the tetrahedral Unit A, it consists of four nodes: 1, 2, 4, 5; for the tetrahedral Unit B, it includes four nodes: 1, 2, 3, 4; for the tetrahedral Units C, it includes four nodes: 1, 2, 3, 6. Then, based on the fixed-solution condition, the linear equations of the potential of the model nodes can be built. The solution conditions are set herein:

The overall energy is minimal. According to the Thomson principle in the electromagnetic field, under boundary and initial conditions, the distribution of the electromagnetic field must meet the minimum energy of the electromagnetic field. Therefore, after obtaining the total energy of the electromagnetic field of the solution region, according to the principle of finding the extreme value, the potential of each node in the solution region can be offset and set to zero.

When the model is set up, the boundary needs to be processed. Therefore, a certain number of node potential is needed to obtain. The total number of nodes called sum_p, the number of known potential nodes known_p, call it kp, Unknown node number unknown_p, called unkp.

(7) $$U=\left[U_{kp}\;U_{unkp}\right]$$

$U_{p}$ denotes the known node potential matrix $U_{f}$ denotes the unknown node potential matrix. Therefore, the total energy is calculated in such a way as to:

(8) $$W=\frac{1}{2}\cdot \left[U_{kp}\;U_{unkp}\right]\times \left(\begin{array}{cc} S_{a} & S_{b}\\ S_{c} & S_{d} \end{array}\right)\times \left(\begin{array}{c} U_{kp}{}^{T}\\ U_{unkp}{}^{T} \end{array}\right).$$

The $U_{kp}{}^{T}$ are known, and $S_{c}$ and $S_{b}$ can be obtained by calculation. Only the $U_{kp}{}^{T}$ are unknown. According to the S matrix property can be obtained:

(9) $$S_{a}U_{unkp}{}^{T}=-S_{b}U_{kp}{}^{T}$$

For easy to calculate, the potential value of the upper interface is set to 10, and the potential value of the lower interface is set to 0. The potential values of all unknown nodes can be obtained by solving the above equations.

**Figure 7:**
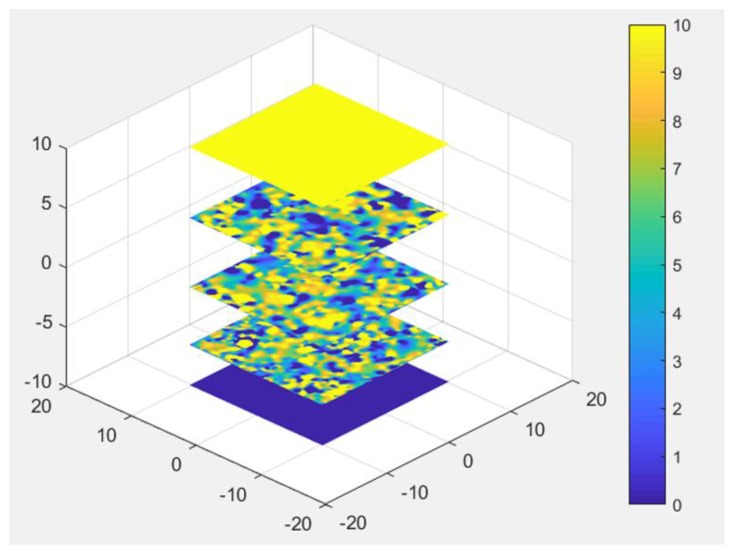
Potential distribution of several cross sections.

The general finite element equation constructed in this paper is a large sparse matrix symmetric algebraic equation group. For solving its solution, a lot of software packages have been used as a solution tool. In this paper, the potential values of all unknown nodes by using the ‘taucs’ software package can be calculated. According to the model established by the proposed, any potential values in the model can be calculated by obtaining the parameters of each node and the interpolation function of each unit. Several cross-sections are selected, and their potential distribution is shown in Figure 7.

The relation between the macroscopic dielectric constant *M* and *W* of a cube is:

(10) $$W=\frac{1}{2}\cdot \varepsilon_{M}LU^{2}.$$

The *U* in the equation represents the voltage at both ends of the plate, and *L* represents the side length of the cube. Therefore, for our three-dimensional model, the expression of macroscopic dielectric constant $\varepsilon_{M}$ is as follows:

(11) $$\varepsilon_{M}=\left\{U_{kp}\times \left(\left[\begin{array}{cc} S_{c} & S_{d} \end{array}\right]\times \left[\begin{array}{c} U_{unkp}{}^{T}\\ U_{kp}{}^{T} \end{array}\right]\right)\right\}/\left(U^{2}L\right)$$

The expected macroscopic dielectric constant epsilon $\varepsilon_{M}$ can be obtained by solving the above equations. 

## 3. Results

**Figure 8:**
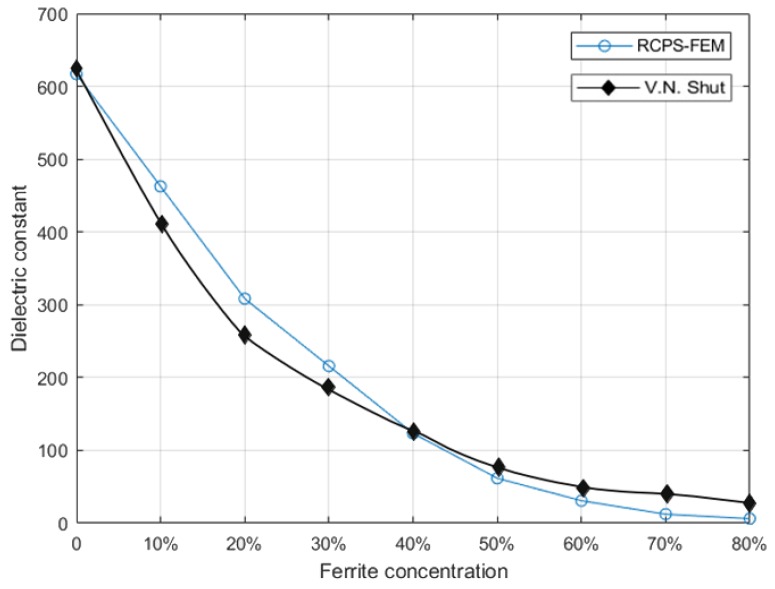
Dielectric permittivity of composites versus ferrite concentration.

According to relevant information, it is known that the permittivity of nickel-zinc ferrite is generally 10–1000, and that of PZT is generally 460–3400 [36,37,38,39,40]. Therefore, the dielectric constant ratios of dual-phase particles are set as 1:500. The results are shown in Figure 8:

According to the above results, it can intuitively see that the larger the volume ratio of Ferrite particle is, the smaller the macroscopic dielectric constant is. Where the black line is Shut, V. N’s experimental result [31].

The simulation results reveal that mathematical method can be a new way to solve the preparation dual-phase composites:

The volume ratio of Ferrite particles less than 20%, dielectric constant of PZT matrix ferroelectric composites decreases significantly. This method can be played a guide to make composites preparation when it is necessary to customize the PZT matrix ferroelectric composites with dielectric constant requirements.

## 4. Conclusions

In this article, a 3D microstructure model of PZT matrix ferroelectric composites is established. And a numerical method is developed to analyze the electrical properties for composites by a three-dimensional microscopic model. The dielectric constant and cross-section potential can be calculated by controlling the volume ratio of the two-phase particles in the model. By building a model of PZT matrix ferroelectric composites, might lead experimenters to have a target for preparation without the unnecessary experimental process.

We come to the following conclusions:

1. According to X-ray (micro-) diffraction microscopy provides 3D microstructural phase representations, and the inputs (such as the number of spheres in unit volume, the duty ratio per unit volume, the radius of the generated sphere and distribution pattern) were obtained by stereological techniques. The inputs can be adjusted to simulate the PZT matrix ferroelectric composites model’s characteristics in different conditions.
2. The ferrite/PZT dual-phase composite model generated by the proposed method is similar to the SEM observation results. This article makes a comparison between simulated particle size and actual particle size (i.e., Figure 1 and Figure 2) to increase persuasion. 3. The 3D model generated by the proposed technique, and the comparison of the effective dielectric constant with experimental and numerical results from the literature (i.e., Figure 8) can be accounted as promising indicators for the resemblance of the model with the real materials.

## References

[1] Lysne P.C. (1977). Dielectric properties of shock-wave-compressed PZT 95/5. J. Appl. Phys..

[2] Mallmann E.J.J., Silva M.A.S., Sombra A.S.B., Botelho M.A., Mazzetto S.E., De Menezes A.S., Almeida A.F.L., Fechine P.B.A. (2015). Dielectric properties of Ca_0.7_Bi_0.3_Ti_0.7_Cr_0.3_O_3_ (CBTC)-CaCu_3_Ti_4_O_12_ (CCTO) composite. J. Electron. Mater..

[3] Chen Y.F., Guo H.Y., Cai M.Z., Geng C.B., Yue C.Y., Teng C.J. (2018). Effect of polyether sulfone resin on micromorphology, thermal, mechanical, and dielectric properties of epoxy-bismaleimide composite material. J. Electron. Mater..

[4] Castro J., de los Rios T., Fuentes L. (2000). Synthesis and characterization of Nb-doped PZT ferro-piezoelectric ceramics. Mater. Manuf. Process..

[5] Guiffard B., Boucher E., Eyraud L., Lebrun L., Guyomar D. (2005). Influence of donor co-doping by niobium or fluorine on the conductivity of Mn doped and Mg doped PZT ceramics. J. Eur. Ceram. Soc..

[6] Salem M.M., Morchenko A.T., Panina L.V., Kostishyn V.G., Andreev V.G., Bibikov S.B., Nikolaev A.N. (2015). Dielectric and magnetic properties of two-phase composite system: Mn-Zn or Ni-Zn ferrites in dielectric matrices. Phys. Procedia.

[7] Wang Z., Wang T., Fang M.R., Wang C., Xiao Y.J., Pu Y.P. (2017). Enhancement of dielectric and electrical properties in BFN/Ni/PVDF three-phase composites. Compos. Sci. Technol..

[8] Inui T., Koga H., Nogi M., Komoda N., Suganuma K. (2015). A miniaturized flexible antenna printed on a high dielectric constant nanopaper composite. Adv. Mater..

[9] Ma P.P., Liu X.Q., Zhang F.Q., Xing J.J., Chen X.M. (2015). Sr(Ga_0.5_Nb_0.5_)_(1-x)_TixO_3_ low-loss microwave dielectric ceramics with medium dielectric constant. J. Am. Ceram. Soc..

[10] Hamar R., Trnka P. Permittivity determination of composite materials based on a 3D electric field model. Proceedings of the International Conference 2016 Conference on Diagnostics in Electrical Engineering (Diagnostika).

[11] Niyonzima I., Jiao Y., Fish J. (2019). Modeling and simulation of nonlinear electro-thermo-mechanical continua with application to shape memory polymeric medical devices. Comput. Methods Appl. Mech. Eng..

[12] Tarasov Y.S., Skripalenko M.M., Radyuk A.G., Titlyanov A.E. (2019). Computer simulation of thermal and stress-strain state of blast furnace tuyeres. Metallurgist+.

[13] Chen X., Moore J.E., Zekarias M., Jensen L. (2015). Atomistic electrodynamics simulations of bare and ligand-coated nanoparticles in the quantum size regime. Nat. Commun..

[14] Andreassen E., Andreasen C.S. (2014). How to determine composite material properties using numerical homogenization. Comput. Mater. Sci..

[15] Nurkanov E.Y., Kadushnikov R.M., Kamenin I.G., Alievskii D.M., Kartashov V.V. (2001). Investigation of the density characteristics of three-dimensional stochastic packs of spherical particles using a computer model. Powder Metall. Met. C+.

[16] Lehikoinen A., Davidsson T., Arkkio A., Belahcen A. A high-performance open-source finite element analysis library for magnetics in MATLAB. Proceedings of the 2018 XIII International Conference on Electrical Machines (ICEM).

[17] Bilotta A., Garcea G., Leonetti L. (2016). A composite mixed finite element model for the elasto-plastic analysis of 3D structural problems. Finite Elem. Anal. Des..

[18] Ko B.H., Kim D., Park N.C., Park Y.P. Study on effective piezoelectric coefficient for finite element analysis of multi-layer ceramic capacitor. Proceedings of the 2015 Joint IEEE International Symposium on the Applications of Ferroelectric, International Symposium on Integrated Functionalities and Piezoelectric Force Microscopy Workshop (ISAF/ISIF/PFM).

[19] Sun Y.X., Qian J.Z., Wang J.L., Zhang M. The calculation of equivalent electromagnetic parameters in multilayer composite materials. Proceedings of the 2017 International Applied Computational Electromagnetics Society Symposium—Italy (Aces).

[20] Isaac F., Marques F., Dourado N., Flores P. (2019). A finite element model of a 3D dry revolute joint incorporated in a multibody dynamic analysis. Multibody Syst. Dyn..

[21] Zhao X.H., Wu Y.G., Fan Z.G., Li F. (2004). Three-dimensional simulations of the complex dielectric properties of random composites by finite element method. J. Appl. Phys..

[22] Guo X.G., Li Y., Jin Z.J., Kang R.K. (2019). Modeling and simulating of high chromium alloy based on molecular dynamics. J. Nanosci. Nanotechnol..

[23] Sheikholeslami M., Jafaryar M., Shafee A., Li Z.X. (2019). Analyze of entropy generation for NEPCM melting process inside a heat storage system. Microsyst. Technol..

[24] Zhou W.B., Wu Y.G. (2009). An effective method to determine the probability density function of spherical second-phase particles. Scripta Mater..

[25] Nersisyan H.H., Yang B.S., Kim B.B., Lee J.H., Won C.W. (2005). Combustion synthesis and characterization of spherical PZT powder. Mater. Lett..

[26] Wensrich C.M. (2012). Boundary structure in dense random packing of monosize spherical particles. Powder Technol..

[27] Labra C., Onate E. (2009). High-density sphere packing for discrete element method simulations. Commun. Numer. Methods Eng..

[28] Niemiec P., Bochenek D., Chrobak A., Guzdek P., Blachowski A. (2015). Ferroelectric-ferromagnetic ceramic composites based on PZT with added ferrite. Int. J. Appl. Ceram. Technol..

[29] Clabel H.J.L., Ferri F.A., Zabotto F.L., Rivera V.A.G., Nogueira I.C., Garcia D., de Lima O.F., Leite E.R., Pereira-da-Silva M.A., Cardoso C.A. (2016). Grain size and interfacial interdiffusion influence on the magnetic and dielectric properties of magnetoelectric La_0.7_Ba_0.3_MnO_3_-BaTiO_3_ composites. J. Magn. Magn. Mater..

[30] Peterson R.C., Jimack P.K., Kelmanson M.A. (1999). The solution of two-dimensional free-surface problems using automatic mesh generation. Int. J. Numer. Methods Fludis.

[31] Shut V.N., Laletin V.M., Syrtsov S.R., Trublovsky V.L., Medvedeva Y.V., Yanushkevich K.I., Bushinskii M.V., Petlitskaya T.V. (2018). Structure, ferroelectric, and magnetoelectric properties of bulk pzt-nife1.9co0.02?(4-) composites. Phys. Solid State+.

[32] Liu Y., Saputra A.A., Wang J.C., Tin-Loi F., Song C.M. (2017). Automatic polyhedral mesh generation and scaled boundary finite element analysis of STL models. Comput. Methods Appl. Mech. Eng..

[33] Yu W.Y., Zhang K., Li X. Recent algorithms on automatic hexahedral mesh generation. Proceedings of the 2015 10th International Conference on Computer Science & Education (ICCSE).

[34] Pochet A., Celes W., Lopes H., Gattass M. (2017). A new quadtree-based approach for automatic quadrilateral mesh generation. Eng. Comput. Ger..

[35] Yamakawa S., Shimada K. (2009). Converting a tetrahedral mesh to a prism-tetrahedral hybrid mesh for FEM accuracy and efficiency. Int. J. Numer. Methods Eng..

[36] Nooman M.A.A., Khan M.N.I., Flossain S.D., Hossain M.F., Samad M.A., Ahmed M.R. (2019). Study the physical, electrical and dielectric properties of calcium doped Ni-Zn ferrites. Mod. Phys. Lett. B.

[37] Bar-haim N., Brunstein M., Grunberg J., Seidamn A. (1974). Electric field dependence of the dielectric constant of PZT ferroelectric ceramics. J. Appl. Phys..

[38] Dong L.J., Xiong C.X., Quan H.Y., Zhao G.H. (2006). Polyvinyl-butyral/lead zirconate titanates composites with high dielectric constant and low dielectric loss. Scripta Mater..

[39] Brockman F.G., Dowling P.H., Steneck W.G. (1950). Dimensional effects resulting from a high dielectric constant found in a ferromagnetic ferrite. Phys. Rev..

[40] Hsiang H.I., Chen T.H. (2009). Electrical properties of low-temperature-fired ferrite-dielectric composites. Ceram. Int..

